# Responses of root physiological characteristics and yield of sweet potato to humic acid urea fertilizer

**DOI:** 10.1371/journal.pone.0189715

**Published:** 2017-12-18

**Authors:** Xiaoguang Chen, Meng Kou, Zhonghou Tang, Aijun Zhang, Hongmin Li, Meng Wei

**Affiliations:** 1 Xuzhou Sweet potato Research Center/ Xuzhou Institute of Agricultural Sciences of the Xuhuai District of Jiangsu Province, Xuzhou, Jiangsu, China; 2 School of Life Science, Jiangsu Nornal University, Xuzhou, Jiangsu, China; Huazhong Agriculture University, CHINA

## Abstract

Humic acid (HA), not only promote the growth of crop roots, they can be combined with nitrogen (N) to increase fertilizer use efficiency and yield. However, the effects of HA urea fertilizer (HA-N) on root growth and yield of sweet potato has not been widely investigated. Xushu 28 was used as the experimental crop to investigate the effects of HA-N on root morphology, active oxygen metabolism and yield under field conditions. Results showed that nitrogen application alone was not beneficial for root growth and storage root formation during the early growth stage. HA-N significantly increased the dry weight of the root system, promoted differentiation from adventitious root to storage root, and increased the overall root activity, total root length, root diameter, root surface area, as well as root volume. HA-N thus increased the activity of superoxide dismutase (SOD), peroxidase (POD), and Catalase (CAT) as well as increasing the soluble protein content of roots and decreasing the malondialdehyde (MDA) content. HA-N significantly increased both the number of storage roots per plant increased by 14.01%, and the average fresh weight per storage root increased by 13.7%, while the yield was also obviously increased by 29.56%. In this study, HA-N increased yield through a synergistic increase of biological yield and harvest index.

## Introduction

Plant roots are the main organ for crops to absorb nutrients and water, and they are the place where physiologically active substances, such as some amino acid and hormones are synthesized. The morphology and physiological characteristics of roots affect growth, development and yield formation of crop [1,2,3,4]. Dysplasia or physiological dysfunction of plant roots will severely affect plant growth and development [5]. One of the main cultivation measures to increase yield of sweet potato is the application of nitrogen; nitrogen affects root growth and differentiation of sweet potato, and ultimately increasing yield [6]. Below a certain range of nitrogen application, the increase of nitrogen into the soil during the early growth stage can increase the total biomass of roots, while root total biomass differentiating to storage roots gradually decreases [7]. Furthermore, a low-level nitrogen application rate (≥ 10mM nitrate) will inhibit root growth [6]. Consequently, the rational use of nitrogen to effectively regulate the balance between absorption and storage functions as well as to realize a synergetic increase of nutrient absorbance and differentiation from adventitious root to storage root are problematic.

Previous studies have shown that humic acid (HA) promotes root growth and the formation of lateral roots, and enlarges the root’s effective absorption area. HA also improves biomass, overall activity and absorption ability of the root system [8,9,10], increases crop carbon and nitrogen metabolism capabilities [11], and promotes differentiation from adventitious root to storage root and increases yield [12]. HA has a strong capability for on complexation and absorption, it easily complexates with urea, and shows significant slow-release effects on nitrogen release and utilization [13,14]. Compared with inorganic fertilizers, HA coated fertilizers convert into plant available nutrients at a slower rate after application, thus making them advantageous by reducing fertilizer use and being less labor intensive. In addition, as decomposed coal is a relatively cheap raw material, HA coated fertilizer can have lower costs. These factors have resulted in an increase in use of HA slow-release fertilizers to gradually become a main research area for new fertilizers in China [15]. Previous studies have shown that HA fertilizer improves fertilizer use efficiency, growth and development of crops (such as potato, maize, and wheat), root activity, dry matter accumulation, yield and quality of crops [13,16]. However, few studies have specifically focused on the effects of HA-N on root morphology, active oxygen metabolism and yield of sweet potato. Therefore, we investigated the effects of HA-N on root biomass, root activity, physiological characteristics of root senescence, and yield formation under field conditions. Results of this study will provide a basis for technical guidance for the reasonable application of HA-N for sweet potato cultivation.

## Materials and methods

### Field design

Field experiments were undertaken from June to October, 2014 and from June to October, 2015 at the Anhui Agricultural University experimental station (33°16′N, 117°45′E), China. Rainfall rate during the sweet potato growing season were 634 mm (2014) and 620 mm (2015). No irrigation was applied either year. Xushu-28,a white-fleshed and widely cultivated sweet potato in China was selected for this experiment. The growing medium was a sandy loam, and the 0–20 cm soil layer contained 1.02% organic matter, 0.54 g kg^-1^ total nitrogen, 30.09 mg kg^-1^ available nitrogen, 18.07 mg kg^-1^ available phosphorus, and 83.26 mg kg^-1^ available potassium. The pH of the soil was 7.83.

The experimental field used in this study belongs to the Anhui Agricultural University, which is a comprehensive research institution, and it has a research ethics review committee to ensure experiments do no harm to crops, animals and humans. Our study was approved by this university, so no specific permissions were required for the described field experiments. The sampling locations were not privately-owned or protected in any way, and this field study did not involve any endangered or protected species. In addition, there was also no vertebrate species in this study.

Five treatments were designed in this study: control (C), humic acid urea treatment (HA-N, completely mixed with weathered coal, HA activator and nitrogen fertilizer, extruding granulation, 16% N, 562.5 kg hm^-2^), weathered coal treatment (HA, 135 kg hm^-2^, humic acid content being equal to that in the HA-N treatment), urea treatment (N, 195.7 kg hm^-2^, nitrogen content equal to that in the HA-N treatment), and humic acid and urea treatment (HA+N, completely mixed with weathered coal, and nitrogen fertilizer, extruding granulation, 16% N, 562.5 kg hm^-2^). Each treatment was replicated three times in a randomized block design. For all treatments, 150 kg hm^-2^ of phosphorus fertilizer and 225 kg hm^-2^ of potassium fertilizer were also applied. All fertilizers were used as base fertilizers. Other management procedures followed standard agricultural practices. Row spacing was 0.8 m and plant spacing was 0.25 m. Plant density was 50,000 plants hm^-2^ with a plot area of 20 m^2^. Sweet potato was planted on June 15 and harvested on October 15, 2014, and planted on June 10 and harvested on October 20, 2015.

The production procedure of HA slow release fertilizer resulted in two features of the HA-coated fertilizer: (1) a specific concentration of sodium hydroxide can increase the activity of weathered coal HA and (2) activated HA can significantly increase the absorption ability of nutrient ions. These features can be used as slow-release mechanisms of HA-coated slow-release fertilizer. Firstly, weathered coal was activated with a specific concentration of sodium hydroxide before being filtered and washed using water, the pH was finally adjusted using ammonia. Subsequent to pH adjusting, the weathered coal sample was mixed with nitrogen for adsorption. The adsorbed sample was then fitted with inorganic fertilizer and granulated via disc granulation, this subsequently being as HA slow-released fertilizer after drying.

The root observation experiment was carried out within the plot experiment. Before ridges were formed in the field, 10 micro-plots were separated using roofing (50 cm height) per treatment. A 30μm nylon net was horizontally tied at the base of each micro-plot to increase water infiltration and root growth. The volume of fertilizer applied, application time and application methods were identical between the experiment plots.

### Sampling and measurements

Root morphology was measured at 40 d and 120 d after planting. All root systems in the soil layer of the separated micro-plot were removed and slowly washed. A 100-mesh sieve was placed under the root system during washing to prevent roots from being washed away. Root total weight was recorded after drying using absorbent paper. Roots from three individual plants having consistent growth were selected and scanned using a root scanner (LA1600+scanner Canada). The WinRHIZO root analytical procedure was used to analyze the scanned root system images.

The root physiological index was measured at 40 d, 80 d and 120 d after planting. 0.5 g of roots was homogenized in 5 cm^3^ of a respective extraction buffer (50 mM phosphate buffered saline (PBS) + 0.4% polyvinylpyrrolidone (PVP), pH 7.0) in a pre-chilled mortar and pestle on ice. The homogenate was centrifuged at 10,000×g for 30 min at 4°C and the supernatant was collected as a crude enzyme extract.

Superoxide dismutase (SOD) activity was assayed by monitoring the inhibition of the photochemical reduction of Nitro Blue Tetrazolium (NBT). One unit SOD activity was defined as the amount of enzyme required to cause 50% inhibition of reduction of NBT as monitored spectrophotometrically (*UV-2401*, *Shimadzu Corp*., Japan) at 560 nm. Activity was expressed as units (U) per gram of fresh root mass (FW).

Peroxidase (POD) activity was determined using the guaiacol oxidation method [17]. Guaiacol oxidation was monitored spectrophotometrically for 60 s at 470 nm. Catalase (CAT) activity was measured by monitoring the decrease in absorbance at 240 nm for 60 s as a consequence of H_2_O_2_ consumption [18]. Malondialdehyde (MDA) was estimated by measuring the content of 2-thiobarbituric acid-reactive substances in a supernatant, prepared in 20% trichloracetic acid containing 0.5% 2-thiobarbituric acid, and heated at 95°C for 25 min. MDA content was then determined spectrophotometrically at 532 nm absorbance and corrected for nonspecific turbidity at 600 nm.

### Yield

All storage roots were harvested and weighted in the yield measure area. Storage roots and plants were counted. The number of storage roots per plant and the average fresh weight per storage root were also calculated.

### Statistical analysis

The analysis of variance was performed with SPSS 18.0 (SPSS Inc., Chicago, USA). Data from each sampling date were analyzed separately. Means were compared using Fisher’s protected least significant difference at P<0.05 (LSD0.05).

## Results

### Effects of HA-N on storage root yield and components

Compared with the C, all fertilization treatments significantly increased storage root yield of sweet potato (Table 1). HA, N, HA+N, and HA-N treatments increased the yield by 5.52%, 6.88%, 21.46%, and 29.56%, respectively (mean value of two years). Compared with the HA, HA+N and HA-N increased yield by 15.1% and 22.78%, respectively. Yield increasing effects of HA-N was significantly better than that of HA+N. Both the number of storage root per plant and the average fresh weight per storage root increased for the HA treatment, however only the results for the HA-N treatment attained a significant level. The treatment which only applied N to the crop resulted in a decrease the number of storage root, although it significantly increased the average fresh weight per storage root. These results indicated that nitrogen increased yield by increasing the average fresh weight per storage root, while HA-N promoted both the number of storage root and the average fresh weight per storage root.

**Table 1:** Yield and yield component factors under different types of nitrogen fertilizer.

Treatments	2014			2015		
	Number of storage root (No.)	Average fresh weight per storage root (g)	Fresh yield (t ha)	Number of storage root (No.)	Average fresh weight per storage root (g)	Fresh yield (t ha)
C	2.71c	205.26c	27.81d	2.50c	202.17b	25.27c
HA	2.84b	195.77c	27.80d	2.73b	206.69b	28.21b
N	2.69c	220.24b	29.62c	2.38c	227.87a	27.11b
HA+N	2.96ab	221.35b	32.76b	2.75b	230.64a	31.71a
HA-N	3.08a	230.39a	35.48a	2.86a	232.85a	33.29a

Means values marked with different small letters indicate significant difference at P = 0.05 levels

### Effects of HA-N on root dry weight and the morphology characteristic index

#### Root dry weight and vine/tuber ratio

The application of fertilizer significantly increased the accumulation of dry matter in sweet potato (Table 2). At the early growth stage, three HA treatments increased dry matter accumulation of root tubers and aerial parts, while they decreased the vine/tuber ratio. Compared with HA and HA-N, HA+N significantly increased dry matter accumulation of root tubers and above ground plant parts, with the effects on above ground plant parts being more apparent. However, N application alone could noticeably decreased the dry weight of storage and absorbing roots, as well as significantly increasing dry matter accumulation of above ground plant parts and the vine/tuber ratio.

**Table 2:** Root and shoot dry matter and vines to tuberous roots of sweet potato.

Years	Treatments	40d				80d				120d			
		Root dry matter (g)	Storage root dry matter (g)	Shoot dry matter (g)	Ratio of vines to tuberous roots	Root dry matter (g)	Storage root dry matter (g)	Shoot dry matter (g)	Ratio of vines to tuberous roots	Root dry matter (g)	Storage root dry matter (g)	Shoot dry matter (g)	Ratio of vines to tuberous roots
2014	C	4.17a	12.20c	34.41c	2.82b	5.16b	34.96d	85.89c	2.46a	4.43c	114.92d	96.68d	0.84a
	HA	4.22a	15.42b	41.19c	2.67c	5.36b	44.68c	97.25b	2.18b	5.08ab	125.25b	105.49c	0.84a
	N	3.94b	9.62d	62.96a	6.54a	5.28b	45.48bc	112.24a	2.47a	4.83b	128.49c	107.47c	0.84a
	HA+N	4.04ab	17.79b	54.34b	3.05b	5.65a	51.26b	106.16b	2.07b	5.12a	133.78b	112.83a	0.84a
	HA-N	3.99b	20.22a	55.88b	2.76bc	5.99a	67.12a	109.83ab	1.64c	5.24a	142.95a	115.41a	0.81b
2015	C	4.42a	14.33b	42.80c	2.99d	5.42c	41.94d	119.72c	2.85a	4.61c	106.14c	93.78d	0.88a
	HA	4.36a	15.22b	69.57ab	4.57b	5.56c	53.77c	98.87d	1.83d	5.21b	121.00b	108.87b	0.90a
	N	4.28a	9.10c	74.54a	8.19a	5.39c	52.93c	135.46b	2.55b	4.75c	132.84b	116.49b	0.88a
	HA+N	4.29a	17.62ab	66.65b	3.78c	5.84b	65.24b	138.46ab	2.12c	5.31ab	144.83ab	129.48a	0.90a
	HA-N	4.32a	20.02a	64.41b	3.22d	6.26a	71.2a	146.87a	2.06c	5.59a	153.67a	134.79a	0.88a

Means values marked with different small letters indicate significant difference at P = 0.05 levels.

Dry matter accumulation of storage root and above ground plant parts recorded a trend of rapid growth following the developmental progress from the fast thickening period to the harvest period. All fertilizer application treatments significantly increased dry matter accumulation of absorbing roots, storage root, and above ground plant parts, and the vine/tuber ratio. The dry weight of absorbing and storage root were in the order of HA-N>HA+N>HA = N. The vine/tuber ratio was in the order of HA-N<HA+N = HA<N.

#### Root morphology characteristics

At the early growth stage, compared with the C, N treatment significantly decreased total root length, root diameter and root surface area, while it slightly decreased root tip number and root volume (Table 3). HA and HA+N treatments increased root diameter, decreased total root length, root tip number, root surface area, and root volume. HA-N treatment significantly increased root diameter and root surface area. These results indicate that the application of HA-N enlarged roots, and promoted the differentiation from adventitious root to storage root, while the application of nitrogen fertilizer alone resulted in a reduction of root thickness and ultimately inhibited root differentiation. At the harvest period and compared with the C, all fertilizer application treatments increased total root length, root diameter, root tip number, root surface area and root volume to different extents; the effects of HA+N and HA-N treatments were maximal.

**Table 3:** Root morphological and physiological characteristics of root (2014).

Treatments	Early growing stages(40d)					Harvest stages(120d)				
	TRL (cm)	RD (mm)	TRN (No.)	TRSA (cm^2^)	TRV (cm^3^)	TRL (m)	RD (mm)	TRN (No.)	TRSA (cm^2^)	TRV (cm^3^)
C	1307a	0.70c	1452a	456.7b	26.42a	923d	0.91c	882c	322.8d	17.28d
HA	1204ab	0.91b	1249b	412.13b	26.58a	1168c	0.98b	1084b	368.4c	18.96bc
N	846c	0.62c	1106c	308.86d	25.38b	1117c	0.94bc	896c	364.6c	18.15c
HA+N	987c	1.01ab	1308ab	364.7c	25.54b	1219b	1.31a	1448a	488.3b	19.62b
HA-N	1182b	1.08a	1426a	516.3a	26.33a	1385a	1.28a	1554a	603.4a	24.71a

TRL-Total root length; RD-Diameter of root; TRN-Total root number; TRSA-Total root surface area; TRV-Total root volume.

Means values marked with different small letters indicate significant difference at P = 0.05 levels

### Effects of HA-N on root vigor and soluble protein content

#### Root vigor

Root vigor, an index reflecting the nutrient absorption efficiency of plants, attained its maximum value for each treatment 80 d after transplantation (Table 4). Compared with the C, all four fertilizer application treatments were beneficial for increasing root vigor. At the early growth stage, root vigor was in the order of HA+N = HA-N>N>HA. From the fast thickening period to the harvest period, root vigor was in the order of HA-N = HA+N>HA>N. These results indicated that HA-N was beneficial to increase root vigor, while retaining increased root physiological activity.

**Table 4:** Root vigor and soluble protein content under different types of nitrogen fertilizer.

Years	Treatments	Root vigor(ugg^-1^h^-1^Fw)			Soluble protein content(mgg^-1^FW)		
		40d	80d	120d	40d	80d	120d
2014	C	77.13c	182.11d	68.46b	0.87c	1.42d	0.93c
	HA	85.01b	207.23bc	72.61b	1.09ab	1.84b	0.97c
	N	90.96ab	198.44c	71.58b	1.14a	1.68c	0.95c
	HA+N	95.58a	217.57b	82.49a	1.13a	1.76b	1.21b
	HA-N	87.35b	246.56a	80.74a	0.98b	1.99a	1.42a
2015	C	71.41c	211.57c	66.74c	0.99c	1.29d	0.94c
	HA	81.20ab	232.70b	78.08b	1.08b	1.74bc	1.10b
	N	83.72a	216.24c	70.19c	1.19a	1.64c	1.05b
	HA+N	82.98a	236.86b	77.62b	1.17a	1.82b	1.23a
	HA-N	77.04b	259.74a	92.04a	1.03bc	2.05a	1.31a

FW-fresh weight.

Means values marked with different small letters indicate significant difference at P = 0.05 levels

### Soluble protein

At the early growth stage, and compared to the C, N, HA, HA+N treatments, and the HA-N treatment increased the soluble protein content by 25.27%, 16.67%, 23.66% and 8.06% in roots, respectively, while the N treatment had the largest growing rate(Table 4). From the fast thickening period to the harvest period, root soluble protein content was in the order of: HA-N>HA+N>HA>N>C. These results indicated that nitrogen fertilizer was beneficial to the synthesis of soluble proteins at the early growth stage, while HA-N significantly increased soluble protein content at the middle and late thickening stage of storage root, thus delaying root senescence.

### Effects of HA-N on antioxidant enzyme activity and MDA content in root system

**Fig 1:**
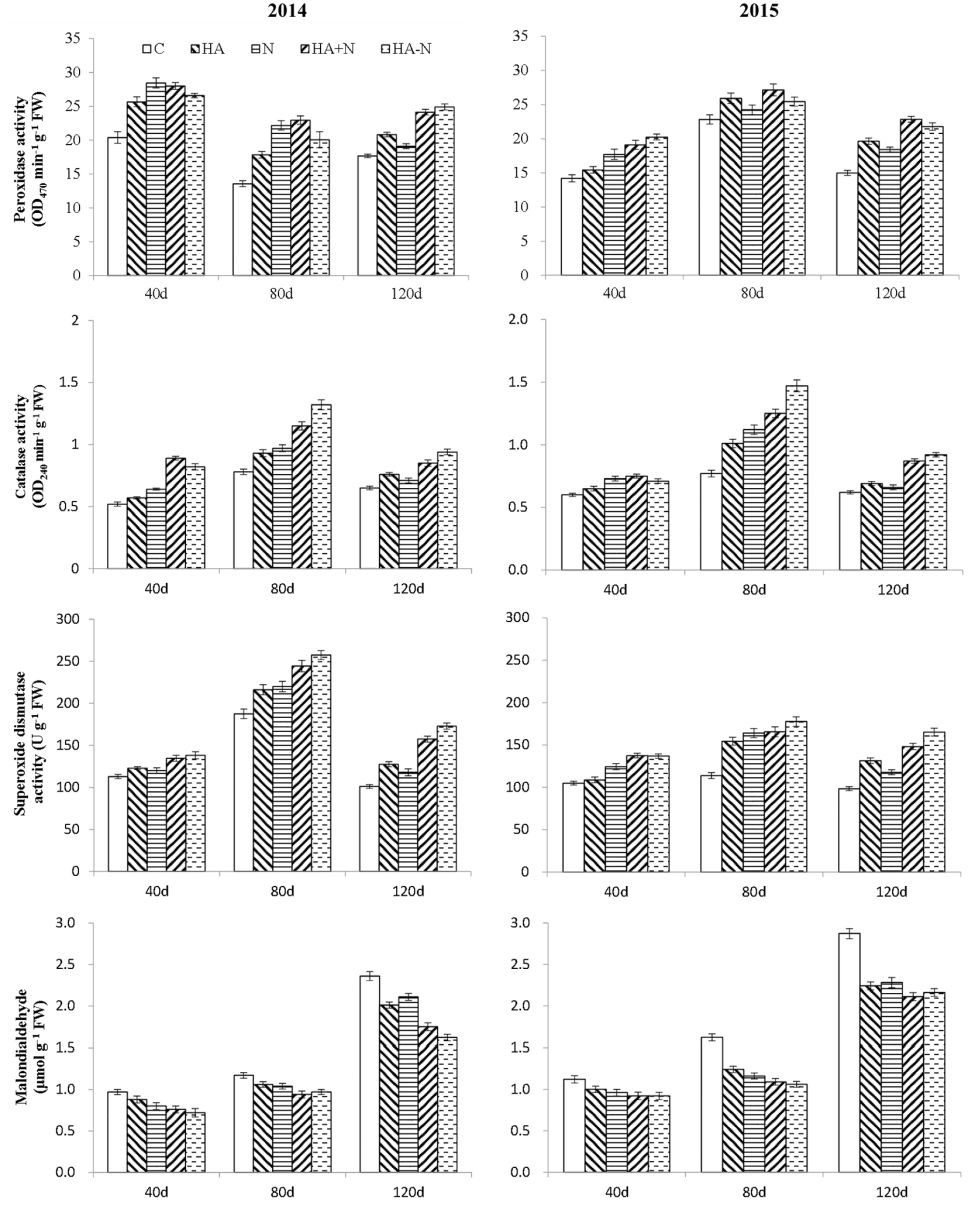
Activities of SOD, POD, CAT and MDA content under different types of nitrogen fertilizer.

From the early to the fast thickening period of storage root, POD activity was highest when subjected to N treatment, being significantly higher compared to the C and HA treatments. However, there was a non-significant difference between HA-N and HA+N. At the harvest period, POD activity was higher when subjected to HA-N treatment and significantly higher compared to the HA and N treatments; however, there was a non-significant difference compared with the HA+N treatment (Fig 1).

CAT activity in sweet potato roots initially recorded an increase before decreasing; CAT activity attained a maximum value 80 d after planting. Compared with the C, CAT activity was increased to a different extent under different fertilizers. CAT activity was significantly higher under HA-N and HA+N treatments, results which indicated that HA-N was beneficial for the elimination of H_2_O_2_ in roots, and thus prevented the root system from aging prematurely.

SOD activity results were similar to those of CAT activity under the different treatments. Compared with the C, all four fertilizer treatments increased SOD activity in roots. SOD activities subjected to HA-N and HA+N treatments were significantly higher compared with the HA and N treatments. However, the difference of SOD activity between HA+N and HA-N treatments was not significant.

MDA content gradually increased after planting with a slow increase for HA-N treatment and a fast increase for the C treatment. Compared with the C treatment, all fertilizer treatments decreased MDA content. In the four fertilizer treatments, MDA content was the lowest for the HA-N treatment, followed by the HA+N treatment; MDA contents were the highest for the HA and N treatments.

### Correlation between root characteristics and yield

Correlation analysis between root morphological indices and yield revealed that root diameter had a significantly positive correlation with yield and storage root number per plant (correlation coefficients were 0.88 and 0.97, respectively) at the early growth stage. Root diameter, root tip number, root surface area, root volume and root activity had either significant or extremely significant positive correlations with yield (correlation coefficients were 0.89, 0.88, 0.96, 0.83 and 0.86, respectively) during the harvest period. As a result, higher root tip number, root surface area, root volume and root activities were maintained at later thickening periods of sweet potato, an occurrence which delayed the rate of root senescence and played an important role in increasing yield.

## Discussions

### The relationship between root morphological-physiological characteristics, yield and components of sweet potato

Plant root morphology and physiological characteristics were closely correlated with growth and development of the above ground plant parts and yield formation. Active roots can provide sufficient nutrients, water and plant hormones for the growth of above ground plant parts to consequently promote the biological yield. Conversely, active above ground plant parts can provide sufficient carbohydrates for transportation to the roots, and promote the activity of root functions [19,20]. Previous studies have shown that root biological yield was closely correlated with that of the above ground plant part [21,22]. For example, a large volume of roots, coupled with their strong absorption ability in the upper layer of rice had a significantly positive correlation with yield [23]. By using established mathematical regression models, it has been indicated that yield can be increased by increasing root length and weight, while retaining a relatively low number of roots. However, when root length and weight increased to a certain extent, yield decreased with an increase of root biomass [24], results which indicate that root development could affect final yield formation. Root length, volume, surface area and the number of root tips were the main morphological indices that reflect root development [25]. Higher total root length, surface area, volume and activity ensured stronger root absorption ability, promoted the formation of effective ears in rice and positively affected rice yield [22, 25, 26].

Compared with other crops, the root structure of sweet potato features a particularity that is not only the organ for nutrient absorption, but also the storage organ of photoassimilates [7,27,28,29]. The growth characteristic of sweet potato is to initially elongate before expanding. When the adventitious roots are elongated to a certain extent, a particular region near the root tip is gradually expands. Numerous lateral roots constantly grow during the growth process of adventitious roots, and roots which develop into absorbing roots and there by promote the absorption and utilization of water and soil nutrients [30]. Furthermore, the growth and development of lateral roots determines the differentiation and formation abilities from adventitious roots to storage root [28,29]. The decrease of total root length, root surface area, and root volume results in a reduction of sweet potato yield. Promoting root development and increasing root surface area and volume can promote nutrient absorption and dry matter accumulation in sweet potato [31]. The increased activity of the absorbing roots ultimately promotes the transfer and accumulation of nutrients and photosynthetic products into storage root, thus increasing yield [31,32]. The results of this study showed that root diameter had a significantly positive correlation with the number of storage root at the early stage of storage root formation. At the late growth stage, root tip number, root surface area, root volume and root activity significantly affected yield. Increased root tip numbers, enlarged root surface area and root volume, as well as higher root activity, ensured that the roots had a stronger nutrient absorption ability, which had a further positive effect on crop yield.

### Effects of HA-N on root morphological-physiological characteristics and yield

Nitrogen was one of the main factors affecting sweet potato growth; it also plays a central role in root growth and construction, and is closely related to the differentiation and formation of storage root [28,32]. Low amounts of nitrogen application presented inhibiting effects on root growth [6]. Within a certain range of nitrogen application, root total biomass increased with increased nitrogen application rate at the early growth stage of plants, while root total biomass differentiating to storage root gradually decreased [7]. An insufficient nitrogen supply led to small and fine sweet potato roots, these not being conducive to root differentiation and storage root formation, consequently decreasing yield [33,34]. However, excessive nitrogen application also had adverse effects on the differentiation and formation of storage root, delayed tuberization and was not conducive to yield [32]. The results of this study showed that nitrogen application alone decreased dry mass of storage root and of the absorbing roots, as well as total root length, root diameter, and root surface area at the early stage of storage root thickening, and reduced the number of storage root. Dry matter accumulation in above ground plant parts and in the vine/tuber ratio were increased, as well as total root length, root diameter, root tip number, root surface area, root volume, and root activity at the harvest stage. Therefore, HA-N treatment significantly increased root diameter, root surface area, the number of storage root, and root activity, as well as promoting dry matter accumulation of above ground plant parts, roots and storage root, and improved the fresh weight per storage root compared with N application alone. Compared with the N and HA+N treatments, when dry weight, total length, surface area and volume of roots all strongly increased, root activity and yield were much higher under the HA-N treatment. This indicated that HA-N is beneficial for the promotion of root growth of sweet potato, it aids the number of storage root and maintain root activity at the late growth stage, promotes dry matter accumulation in storage root, and increases yield.

### The effects of HA-N on the active oxygen metabolism

Senescence of plants, the accumulative process of metabolic disorders of active oxygen and free radicals [35], is closely related to an active oxygen metabolism. The coordinated function of antioxidant enzymes, such as SOD, POD and CAT, effectively eliminates active oxygen free radicals [36,37]. The results of this study revealed that nitrogen application alone and HA-N application could effectively increase the activities of SOD, POD and CAT, decrease MDA content, and significantly increase soluble protein content compared with no fertilizer added. However, nitrogen application alone strongly affected activities of anti-senescence enzymes at the early growth stage, and HA-N strongly promoted antioxidant enzyme activities in roots during the whole growth stage, especially leading to significant effects at the late growth stage. This finding suggests that by retaining higher activities of protective enzymes, thus eliminating active oxygen in time, HA-N decreased preoxide levels, relieved membrane damage, delayed root senescence, and increased mineral nutrient absorption ability of roots.

## Conclusions

HA-N effectively promoted the differentiation from adventitious root to storage root at the early growth stage, as well as increasing storage root numbers per plant. HA-N increased yield through a synergistic increase of biological yield and harvest index. Higher biomass, activity, absorbing area and volume of roots, as well as higher anti-ageing enzyme activities, promoted nutrient absorption as well as aboveground and underground biomass accumulation of sweet potato. This was the physiological basis for the observed yield increase of HA-N.

## Supporting information

S1 Dataset S1 Dataset contains data on activities of SOD, POD, CAT and MDA content data (Fig 1). (XLSX)
